# Molecular and antigen tests, and sample types for diagnosis of COVID-19: a review

**DOI:** 10.2217/fvl-2021-0256

**Published:** 2022-07-01

**Authors:** Yujia Zhang, Rachael Garner, Sana Salehi, Marianna La Rocca, Dominique Duncan

**Affiliations:** 1 Laboratory of Neuro Imaging, USC Stevens Neuroimaging & Informatics Institute, Keck School of Medicine of USC, University of Southern California, 2025 Zonal Ave., Los Angeles, CA 90033, USA; 2 Università di Bari, Dipartimento Interateneo di Fisica, Bari, Italy

**Keywords:** COVID-19, diagnostic testing, SARS-CoV-2, sensitivity and specificity, US FDA

## Abstract

Laboratory tests seeking to improve detection of COVID-19 have been widely developed by laboratories and commercial companies. This review provides an overview of molecular and antigen tests, presents the sensitivity and specificity for 329 assays that have received US FDA Emergency Use Authorization and evaluates six sample collection methods – nasal, nasopharyngeal, oropharyngeal swabs, saliva, blood and stool. Molecular testing is preferred for diagnosis of COVID-19, but negative results do not always rule out the presence of infection, especially when clinical suspicion is high. Sensitivity and specificity ranged from 88.1 to 100% and 88 to 100%, respectively. Antigen tests may be more easy to use and rapid. However, they have reported a wide range of detection sensitivities from 16.7 to 85%, which may potentially yield many false-negative results.

## Global pandemic

On 31 December 2019, several unknown pneumonia cases were reported to the WHO. Later, the cause of those cases was confirmed as the 2019 novel coronavirus (2019-nCoV) and renamed as SARS-CoV-2 [1]. People who acquire COVID-19 experience variable outcomes: some are asymptomatic or recover from minor influenza-like symptoms without requiring any treatment; however, others develop severe illnesses such as respiratory failure. Severe outcomes are especially common at advanced ages and in people with underlying medical conditions [2]. COVID-19 has become a global challenge because it can spread easily and rapidly through respiratory droplets [3]. Working and living conditions for citizens worldwide changed significantly to curb the spread of disease [4]. Hence, it is critical to accurately detect the presence of the virus to control the disease transmission.

## Motivations & objectives

There are three types of tests for COVID-19: molecular, antigen and antibody tests [5]. Various sample collection methods have also been utilized for each type of test assay. The goal of this paper is to present the sensitivity and specificity of 329 commercial and laboratory-developed assays and evaluate six sample collection methods.

## Structure & replication of SARS-CoV-2

**Figure 1.**
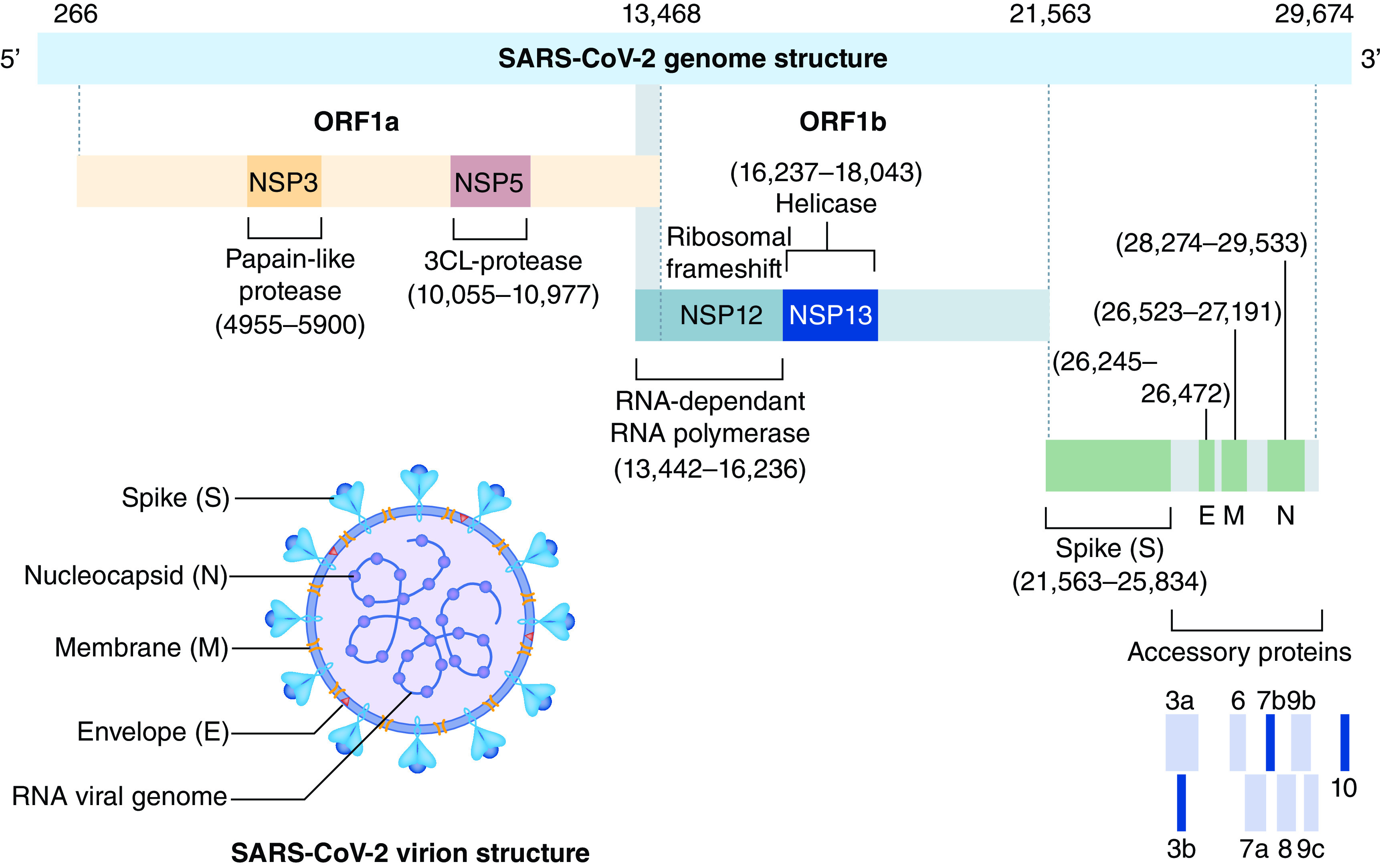
Genomic structure of SARS-CoV-2. NSP: Nonstructural proteins; ORF: Open reading frames. Reproduced from Alanagreh *et al.* with permission from [7] under a CC BY 4.0 license (https://creativecommons.org/licenses/by/4.0/).

Coronavirus (CoV) genomes contain six open reading frames (ORFs) that can encode proteins [6,7]. Two ORFs (ORF1a/b) near the 5′-terminus compose two-thirds of the genome length and encode polyproteins PP1ab and PP1a that eventually split into 16 nonstructural proteins. The remaining ORFs near the 3′-terminus make up the other a third of the genome length and encode four major structural proteins: S, M, E, N and accessory proteins (Figure 1) [8].

**Figure 2.**
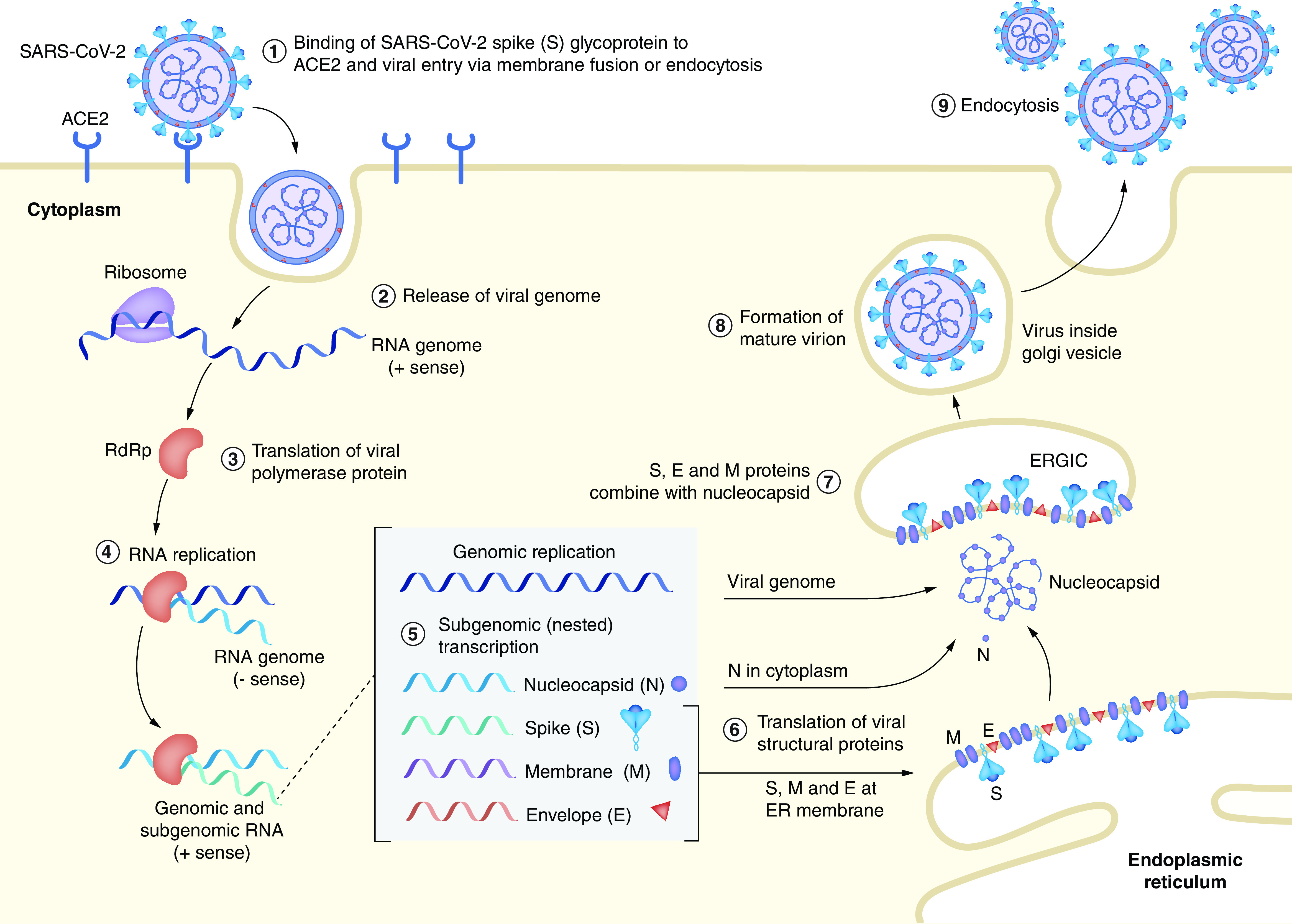
The infection and replication cycle of SARS-CoV-2. ERGIC: Endoplasmic reticulum–Golgi intermediate compartment. Reproduced from Alanagreh *et al.* with permission from [7] under a CC BY 4.0 license (https://creativecommons.org/licenses/by/4.0/).

The S proteins initiate host cell invasion by SARS-CoV-2 via binding it to a surface receptor protein, ACE2, which is the entry route for the coronavirus into host cells [9,10]. M proteins play a significant function by stimulating the virions and the E proteins permit adhesion to the membrane of viruses, pulling together and promoting morphogenesis of virions inside of the cells. The N proteins are critical for replication and transcription of viral genomic RNA [10]. SARS-CoV-2 has 8 accessory proteins: 3a, 3b, 6, 7b, 8b, 9b and ORF14 [6]. During the transcription process, groups of subgenomic RNAs are produced, and after that, the virions are released from the infected cell through exocytosis (Figure 2) [7].

## Tests for COVID-19 detection

Currently, there are two major categories of COVID-19 detection toolkits: molecular tests and antibody/serology tests [11]. Molecular tests detect viral genomic RNA, which only remains in the human body while the virus is actively replicating. Antibody tests detect specific antibodies produced by the human immune system in response to the infection [12]. Although antibody tests have high levels of specificity to indicate whether people have had an active immune response to the SARS-CoV-2 antigen, antibodies may not be detectable until 1–3 weeks after infection [3]. Also, vaccination results in a positive spike antibody and may not be a reliable method of testing in vaccinated individuals. Hence, antibody testing will not be discussed in detail in this paper.

## Materials & methods

A comprehensive online literature and data search was conducted using the keywords ‘COVID-19 test assay’, ‘diagnostic kits’ and ‘COVID-19 detection’ on Google Scholar and Embase (Elsevier), and the results were screened by authors. This study contains 45 peer-reviewed articles related to COVID-19 test and sample collection techniques as well as data on the sensitivity and specificity of each assay. Sensitivity refers to the ability of a test to correctly identify patients with a disease (true positive rate), and specificity is the ability of a test to correctly identify patients without a disease (true negative rate) [13]. There are likely differences between sensitivities and specificities of each test in clinical testing and real-world environments. However, actual performance metrics obtained under real-world conditions are not well documented or published by the test manufacturers. Therefore, all sensitivities and specificities reported in this manuscript are those stated in Emergency Use Authorization (EUA) reports published by the US FDA.

## Diagnostic testing

Diagnostic testing of COVID-19 has become an important area of study for researchers due to the urgent need to detect the disease to curb its spread and the rapid evolution of diagnostic strategies. Since 15 March 2020, hundreds of COVID-19 diagnostic tests have received EUA from the FDA.

### Molecular tests

#### Reverse transcription-PCR

Molecular testing usually targets multiple viral genes such as the RNA-dependent RNA polymerase-encoding (*RdRp*) gene and the viral *N* gene [14]. The PCR method is one of the most reliable and widely used methods to rapidly detect viruses due to its high sensitivity and specificity [15]. PCR synthesizes a strand of DNA complementary to a template strand, while reverse transcription PCR (RT-PCR) uses RNA as a template. RT-PCR is the main method to detect SARS-CoV-2 because it can adequately diagnose COVID-19 even in the early stages of infection with high specificity [15]. RT-PCR is used for detection by amplifying specific genomic sequences of viruses [16].

**Figure 3.**
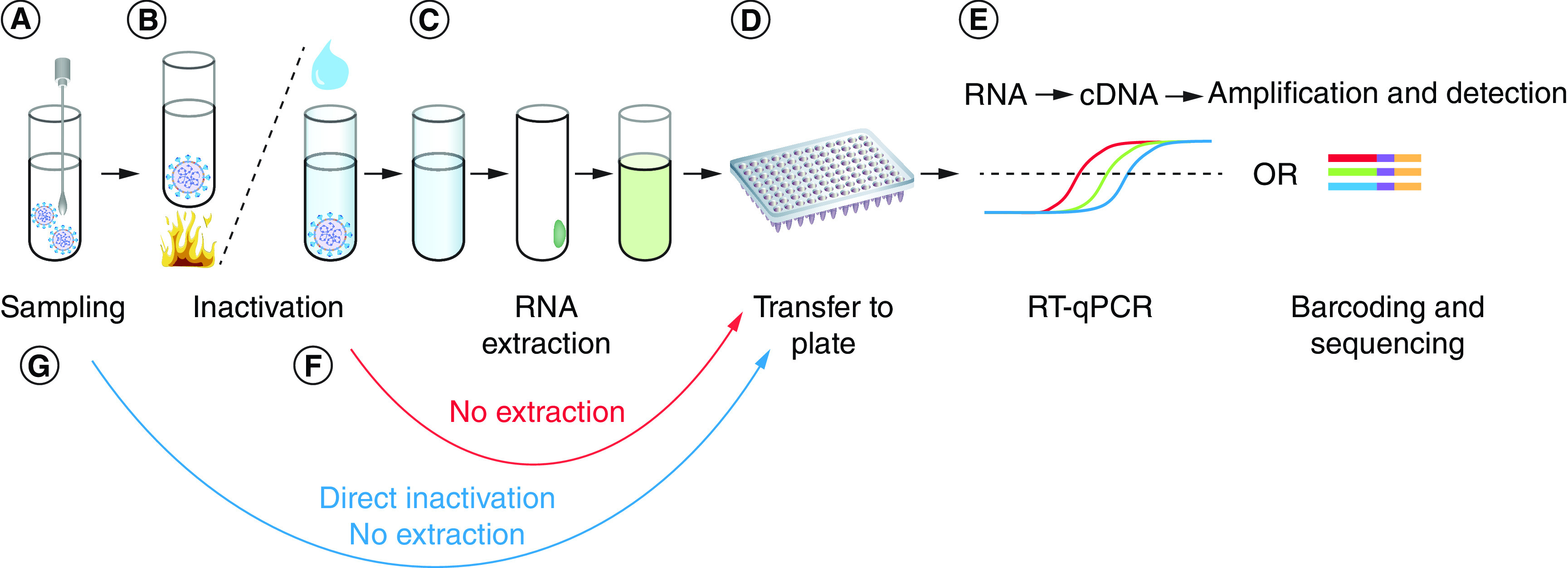
A summary of the RT-PCR amplification method. RT-PCR: Reverse transcription-PCR; RT-qPCR: Reverse transcription-quantitative PCR. Reproduced from Smylaki *et al.* with permission from [17] under a CC BY 4.0 license (https://creativecommons.org/licenses/by/4.0/).

The method is simple to apply in a laboratory setting. After the viral RNA is isolated and purified from sample specimens, reverse transcriptase catalyzes the formation of cDNA using RNA as a template (Figure 3) [11,17].

Although RT-PCR is a preferred technique for diagnosis of COVID-19, the negative results do not always rule out the disease especially when clinical suspicion is high [18]. Prevalence of a disease, a key parameter of pretest probability, impacts the positive and negative predictive value. Repeated testing may be required since false-negative results are more prevalent in areas with high pretest probability [19–21]. Supplementary Table 1 reports a list of 244 authorized RT-PCR tests that have received EUA from the FDA for COVID-19 detection (Supplementary Table 1) [22]. The sensitivities and specificities of 173 assays were reported to range from 88.1 to 100% and 90.9–100%, respectively.

#### Reverse transcription-quantitative PCR

Quantitative RT-PCR (RT-qPCR) can also be used for COVID-19 diagnosis due to its high sensitivity. However, it is less widely utilized because it requires expensive instrumentation [23]. RT-qPCR combines the RT-PCR technique with real-time quantification of amplified DNA using fluorescent probes. After collecting data and isolating the RNA, RT-qPCR follows the same method to detect the viral genome by transcribing the viral RNA to DNA, followed by the semiquantitative DNA PCR [23]. Some tests provide a way to measure viral load in the sample, known as the cycle threshold (Ct) value. Ct cutoff values are relative and specific to each test protocol, but generally indicate the number of PCR cycles required for adequate viral detection (e.g., 35–40 cycles). At the Ct point, the qPCR fluorescence exceeds background fluorescence levels and the test is regarded as positive [24]. Ct is inversely proportionate to the amount of viral nucleic acid in the sample – low Ct values indicate high viral load in the sample, therefore fewer cycles are required for sufficient amplification for detection. In assays with high Ct cutoffs, the protocol allows for a greater number of PCR cycles to yield sufficient viral replication. Therefore, more samples will test positive, increasing the sensitivity of the test, but at the same time, likely reducing the specificity [25]. This poses challenges for those who recover from COVID-19, as positive RT-qPCR results have been reported even after the live virus was no longer detectable [26]. Concerns have also been raised in the context of disease onset when a sample’s Ct value may be higher than the cutoff range at the time of testing but may decrease to within-range values as the disease course progresses. For clinical application, Ct values may therefore be more useful when assessed in the context of symptom onset and resolution [24].

The specific implementation of RT-qPCR varies from test center to test center based on the volume of samples and processing capabilities. Overall, it is relatively time consuming: usually taking 3–24 h [23]. Also, since the onset of the COVID-19 pandemic, the supply chain that supports the manufacturing and distribution of reagents has been impacted by high demand and import/export restrictions. Thus, reliable replenishment of supplies is not always available.

Supplementary Table 2 reports a list of four authorized RT-qPCR tests for COVID-19 detection that have received EUA from the FDA [22]. The sensitivities and specificities of three assays were reported to range from 97.1 to 100% and 98.2 to 100%, respectively.

#### Reverse transcription loop-mediated isothermal amplification

Reverse transcription loop-mediated isothermal amplification (RT-LAMP) is a faster method for genome amplification and can be applied to detect the presence of SARS-CoV-2. RT-LAMP can be accomplished in a single, 20-min protocol with incubation at a constant temperature of 65°C, unlike PCR, which requires a multi phased protocol where each step occurs at a temperature optimal for catalysis [27]. RT-LAMP reactions include reverse transcriptase, DNA polymerase with strong strand displacement activity and tolerance for elevated temperatures, and up to six DNA oligonucleotides of a certain genomic structure [23]. There are various approaches to detect DNA production in RT-LAMP assays, such as a pH indicator. Technicians can run the reaction in a weakly buffered environment, monitoring for pH changes that occur as the reaction proceeds, indicated by a color change [23]. Overall, this technique provides a rapid and accessible way for viral detection. Supplementary Table 3 reports a list of eight authorized RT-LAMP tests for COVID-19 detection that have received EUA from the FDA [22]. The sensitivities and specificities of six assays were reported to range from 95 to 100% and 100%, respectively.

#### Next-generation sequencing

**Figure 4.**
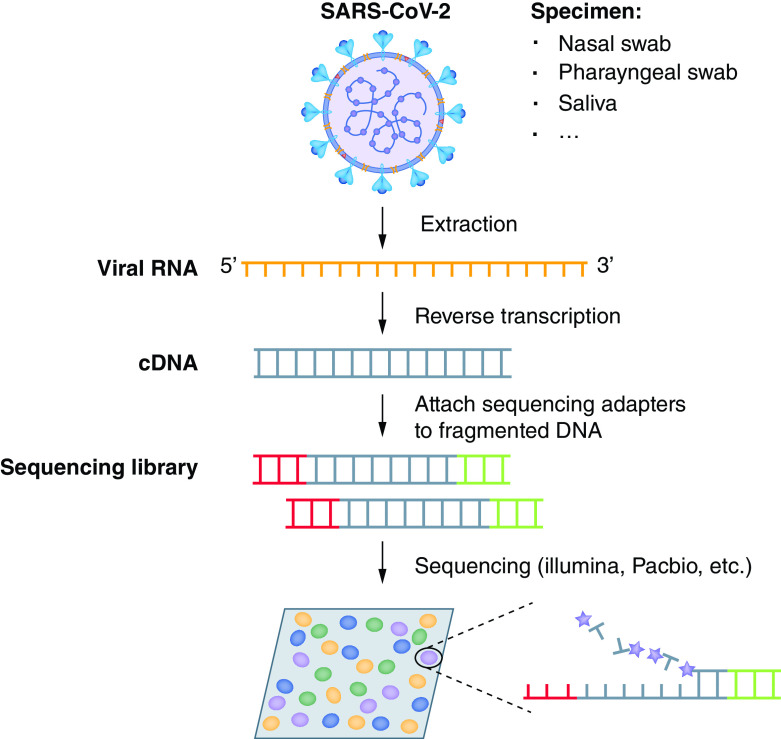
Next-generation sequencing. Reproduced from Xu *et al.* with permission from [29] under a CC BY 4.0 license (https://creativecommons.org/licenses/by/4.0/).

Next-generation sequencing (NGS) is used for the Phylogenetic Assignment of Named Global Outbreak (PANGO) lineages of SARS-CoV-2 [28]. It is a highly scalable, although expensive, genomic analysis method that allows researchers to sequence thousands to billions of DNA fragments in a single run (Figure 4) [29]. The method allowed for rapid comparison of the SARS-CoV-2 to other viral sequences, illuminating nearly 80% genomic similarity with SARS-CoV, which was first reported in Asia in February 2003, and 96% similarity to a coronavirus that affects bats [29]. The sequencing reactions occur in parallel on a solid surface such as glass or beads and contain several steps: nucleic acid (DNA/RNA) extraction, NGS sequencing (targeted sequencing, whole-exome sequencing and whole-genome sequencing), library generation (DNA fragmentation, ligation of adaptors, amplification and sample enrichment), cluster/template generation, sequencing and evaluation of sequence [24]. Supplementary Table 4 reports a list of six authorized NGS tests that received EUA for COVID-19 detection from the FDA [22]. The sensitivities and specificities of six assays were reported to range from 90 to 100% and 97.4 to 100%, respectively (Supplementary Table 4).

#### Clustered regularly interspaced short palindromic repeats

**Figure 5.**
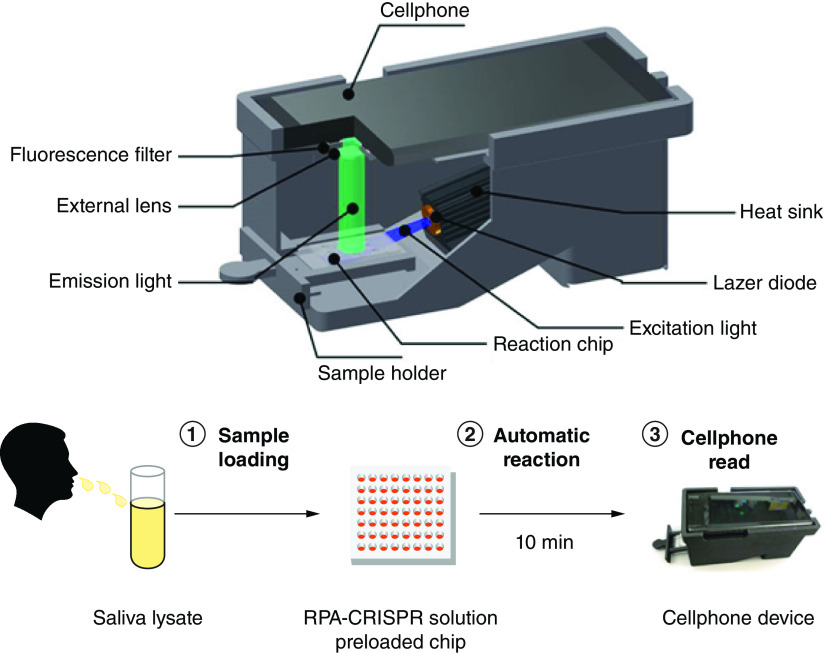
Schematic of smartphone-based CRISPR assay reader. CRISPR: Clustered regularly interspaced short palindromic repeats. Reproduced from Ning *et al.* with permission from [36] under a CC BY 4.0 license (https://creativecommons.org/licenses/by/4.0/).

CRISPR was first introduced by groups of researchers and bioengineers in 2017 and was shown to be useful for detecting Zika and Dengue virus in 2018 [30,31]. It is another promising innovative method for COVID-19 detection and it is as efficient as RT-qPCR when detecting asymptomatic patients [32]. This technology is designed for high specificity, high precision, high efficiency and easy operation [29]. The protocol is low-cost and typically only requires 5–20 mins [33]. It involves capturing and inserting foreign DNA fragments into a bacterial genome, after which, Cas endonuclease is guided to remove the foreign nucleic acid [29,34]. RT-LAMP is then used to target viral genomic RNA and reverse transcribed RNA to DNA and then DNA is amplified by a strand-displacing DNA polymerase (Figure 5). Next, the transcription of the amplified DNA activates the collateral cleavage activity of a CRISPR complex programmed to the target RNA sequence [35]. A study has shown that the sensitivity of CRISPR assays ranged from 93.8 to 100% and the specificity for CRISPR assays ranged from 88 to 100% [34,36]. Supplementary Table 5 reports a list of two authorized CRISPR tests that have received EUA for COVID-19 detection from the FDA [22]. However, the sensitivity and specificity of those two authorized CRISPR tests have not yet been reported.

#### Transcription-mediated amplification

Transcription-mediated amplification (TMA) is another method for nucleic acid amplification testing. TMA has become one of the most sensitive detection assays for HCV, and it can be also used in COVID-19 detection by isothermally amplifying rRNA using reverse transcriptase and RNA polymerase [37,38]. Its main benefit over RT-PCR is that it does not require energy- and time-intensive thermal cycling. TMA uses reverse transcriptase to make DNA from RNA. The original viral RNA is degraded by reverse transcriptase as the cDNA strand is formed, removing the need for a high-temperature denaturing step. In combination with probe hybridization, TMA can be used to isolate, amplify and detect the internal process to control RNA and unique sequences [39]. A recent study demonstrated the higher analytical sensitivity of TMA compared with RT-PCR. It showed that the sensitivity and specificity of TMA for SARS-CoV-2 are 98 and 100%, respectively. TMA provided fewer inconclusive results compared with RT-PCR [40]. TMA is also automated and can support high-volume analysis, potentially more than 1000 samples per system can be processed and analyzed per day [39]. Supplementary Table 6 includes a list of seven authorized TMA tests that have received EUA from the FDA [22]. The sensitivities and specificities of seven assays were reported to range from 96.1 to 100% and 98 to 100%, respectively.

### Rapid antigen test

SARS-CoV-2 N proteins are usually the ideal target for antigen-based detection [41]. Compared with PCR-based assay techniques, antigen tests may be more easy to use and rapid [42]. Antigen tests can be used for both asymptomatic and symptomatic patients 5–12 days after symptoms onset and results can be reported within 15 min [43]. Some COVID-19 rapid antigen tests target SARS-CoV-2 nucleoprotein in patient specimens [44]. However, concerns remain surrounding the performance of antigen tests due to low detection sensitivity, which may yield many false-negative results. In realistic scenarios, antigen tests are less sensitive than RT-PCR and less reliable in samples with lower viral load. The sensitivity of antigen tests varies. Recent studies show that antigen tests demonstrated a wide range of sensitivity from 16.7 to 85% [45]. Hence, the WHO recommended that antigen tests be used for research purposes rather than to inform patient care [42]. Therefore, antigen tests are recommended to be used in conjunction with RT-PCR testing, especially in situations with high pretest probability [46,47]. Supplementary Table 7 reports a list of 26 authorized antigen tests that have already received EUA from the FDA [22]. From these 26 antigen tests, sensitivity was reported to range from 83.5 to 100% and specificity ranged from 93.9 to 100%. The sensitivities and specificities of 20 assays were reported to range from 83.5 to 97.7% and 93.9 to 100%, respectively.

### At-home testing

In addition to testing conducted by healthcare workers, at-home testing has emerged to ease and expedite COVID-19 testing. At-home testing is easy to access and perform and the results are usually available within 15–30 min. People can collect their own nasal and saliva specimen samples following the manufacturer’s instructions and communicate with their healthcare providers after receiving results [3].

The majority of at-home rapid tests are antigen tests, and colorimetric detection is one method for at-home antigen testing. Leuco crystal violet is a colorless dye, but when it contacts with double-stranded DNA after reverse transcription from RNA, it turned into crystal violet [48]. At-home collection kits are easily accessible and widely used. They are available either by prescription or over-the-counter [3]. People collect their nasal, nasopharyngeal (NP) or oropharyngeal (OP) swabs at home and then send samples to labs for processing.

The advantage of at-home testing is that patients can avoid traveling between test centers because there is always a danger of infection transmission to patients, visitors and healthcare workers [49]. Although those tests can provide accurate positive results in symptomatic patients, negative results are less reliable than molecular tests performed in the laboratory. Not all of at-home testing are authorized for asymptomatic patients [49]. Furthermore, COVID-19 molecular tests are available to everyone in the USA with low or no costs; however, at-home tests are often sold by pharmacies or retailers that cost higher than molecular tests. As the pandemic goes on, especially during surges in number of new cases, demands for at-home testing are increasing and testing kits become scarce.

Supplementary Table 8 reports a list of three authorized at-home tests that have already received EUA from the FDA [22]. The sensitivities and specificities of two assays were reported to range from 91.7 to 100% and 98 to 100%, respectively.

Supplementary Table 9 reports a list of 59 authorized at-home sample collection kits that have already received EUA from the FDA [22]. The sensitivities and specificities of 25 assays were reported to range from 95 to 100% and 90.9 to 100%, respectively.

## Sample collection techniques

### Swab tests

The CDC identifies NP swabs, OP swabs and nasal swabs as acceptable upper respiratory sample specimens for SARS-CoV-2 RNA detection [50]. Among these, nasal swabs are reported to have the highest viral titers [51]. Compared with NP swabs, OP swabs cause less discomfort for patients, are easier for collection and require less training by health workers [52]. However, OP swabs may be less sensitive than NP swabs because NP swabs had lower Ct values [52]. Usually, OP swabs, NP swabs and nasal swabs are used in molecular tests; nasal swabs or NP swabs are typically used in antigen tests [5].

### Saliva tests

In addition to swab collections, saliva tests also play an important role in sample collection for molecular tests [53]. Compared with swab samples, saliva is safer for healthcare workers because patients can self-administer to spit saliva instead of receiving more invasive nasal or throat swabs and it is generally regarded as more comfortable for patients. Based on recent clinical findings, the diagnosis efficiency of saliva tests varies from 30.7% up to 100%. The diagnosis efficiency has been shown to be 30.7 and 75% in noncritically ill and critically ill patients, respectively, with the samples collected from the opening of the salivary gland canal. The diagnosis efficiency can reach 100% with the samples collected from drooling saliva [53].

### Additional collection techniques

Blood samples are mainly used for COVID-19 antibody test to detect three types of antibodies including IgG, IgM and IgA which were produced in response to the infection [54]. The main specimen types used for virus detection are those of the upper respiratory tract as described above. Stool sample may also hold utility as an additional source for diagnosis and It has been shown to have SARS-CoV-2 RNA detectable by PCR in some patients for more than 4 weeks [55]. Furthermore, a number of studies also confirmed that the SARS-CoV-2 RNA was present in stool samples for both symptomatic and asymptomatic carriers [56–59].

## Future perspective

Currently, molecular testing is the preferred technique for the diagnosis of COVID-19 due to its high sensitivity and specificity. However, the estimated process time takes longer than expected. Usually, it takes 60–90 min for RT-PCR and 30–45 min for RT-LAMP excluding the time for data analysis or reporting [60]. Also, it is important to reduce the cost of sample collection, testing and analysis [60]. As detection and diagnostic methods innovate over time, those tests will ideally become more time efficient and cost efficient. The COVID-19 pandemic has driven the emergence of more innovative technologies for detection, such as the use of artificial intelligence and machine learning. Matrix-assisted laser desorption ionization-TOF–mass spectrometry is thought to be a very promising new approach due to its low-cost, rapid and high-throughput solution for molecular testing [61]. It can achieve sensitivity and specificity of 100 and 96% compared with PCR [61]. Those innovative solutions will increase the efficiency of identifying patients with COVID-19 in the future.

## Conclusion

This review summarized the molecular and antigen tests for COVID-19 detection, presented the sensitivity and specificity of commercial and laboratory-developed assays, and evaluated sample collection methods. Molecular testing is the most widely used for the diagnosis of COVID-19 due to its high sensitivity and specificity compared to antigen testing.

**Executive summary**

**Molecular tests**

- Molecular testing is the preferred technique for the diagnosis of COVID-19 due to its high sensitivity and specificity, but negative results do not always rule out the presence of infection, especially when clinical suspicion is high.
- The sensitivity of 282 molecular tests ranged from 88.1 to 100% and specificity ranged from 88 to 100%.

**Antigen tests**

- It has been stated in US FDA Emergency Use Authorization reports that detection sensitivity of antigen tests is as low as 83.5% which may yield many false-negative results.
- In practice, antigen tests are less sensitive than reverse transcription-PCR and less reliable in samples with lower viral load.
- Recent studies have shown that antigen tests demonstrated a wide range of sensitivity from 16.7 to 85% in clinical usage.

**At-home tests**

- At-home testing has emerged to ease self-collection of nasal and saliva samples and minimize risks of exposure at testing sites.
- Although at-home test kits can provide accurate positive results in symptomatic patients, negative results are less reliable than molecular techniques performed in the laboratory, especially in asymptomatic patients.
- COVID-19 molecular tests are available to everyone in the USA at low or no costs; however, at-home tests are often sold by pharmacies or retailers that cost higher.

**Sample collection techniques**

- Among nasal, OP and NP swabs, nasal swabs are reported to have the highest viral titers.
- Compared with swab samples, saliva is safer for healthcare workers because patients can self-administer and it is generally regarded as more comfortable for patients.
